# *senX3*-independent contribution of *regX3* to *Mycobacterium tuberculosis* virulence

**DOI:** 10.1186/s12866-014-0265-8

**Published:** 2014-10-25

**Authors:** Dalin Rifat, Deborah A Belchis, Petros C Karakousis

**Affiliations:** Department of Medicine, Johns Hopkins University School of Medicine, Center for Tuberculosis Research, 1551 East Jefferson Street, Room 110, Baltimore, MD 21287 USA; Department of Pathology, Johns Hopkins University School of Medicine, Baltimore, MD 21287 USA; Department of International Health, Johns Hopkins Bloomberg School of Public Health, Baltimore, MD 21205 USA

**Keywords:** *Mycobacterium tuberculosis*, Specificity, Stringent response, Persistence, Virulence, Phosphate, Nutrient, Starvation, Mouse, Cross-regulation

## Abstract

**Background:**

*Mycobacterium tuberculosis* (Mtb) must adapt to various stress conditions during host infection. The two-component regulatory system (2CRS) SenX3-RegX3 is required for Mtb virulence. We showed recently that the *senX3*-*regX3* intergenic region contains promoter activity, driving *senX3*-independent *regX3* expression. In the current study, we tested the hypothesis that RegX3 has a SenX3-independent role in Mtb virulence. The gene expression patterns, growth, and survival of mutants containing transposon insertions in *senX3* (*senX3*::Tn) and *regX3* (*regX3*::Tn) were compared to those of their respective complemented strains and the isogenic wild-type parent strain during axenic growth in nutrient-rich broth, phosphate depletion, nutrient starvation, and in the lungs of BALB/c mice.

**Results:**

*regX3* expression was reduced in *senX3*::Tn during phosphate depletion and nutrient starvation, and expression of the phosphate-specific transport gene *pstC2* was reduced similarly in *senX3*::Tn and *regX3*::Tn during phosphate depletion. Although *senX3* and *regX3* were each dispensable for Mtb growth in nutrient-rich broth, disruption of *senX3* or *regX3* caused a similar growth defect during phosphate depletion. Interestingly, *senX3*::Tn, in which monocistronic *regX3* expression is preserved, showed significantly higher survival relative to *regX3*::Tn after 7 days of nutrient starvation (*p* <0.01), and in mouse lungs at Day 31 (*p <* 0.01), Day 62 (*p* < 0.01), and Day 124 (*p =* 0.05) after aerosol infection.

**Conclusion:**

Our data demonstrate the specificity of the *senX3-regX3* 2CRS for sensing and responding to low ambient phosphate, but also raise the possibility that RegX3 may function independently of its cognate sensor histidine kinase.

## Background

Two component regulatory systems (2CRS) comprise a sensor histidine kinase (HK) and a response regulator (RR), which sense and respond to various environmental conditions, thus promoting bacterial survival [1]. Upon detection of a specific stimulus, the HK autophosphorylates and transfers a phosphoryl group to the cognate RR, which promotes DNA binding of the latter to initiate transcription of the appropriate adaptive response [2,3]. However, under certain circumstances, bacterial RR may also be phosphorylated by a noncognate HK or small molecule acetyl phosphate, merging different stimuli or diversifying the response to a single signal [4], or may function without being phosphorylated [5-8].

Twelve complete pairs of 2CRS and a few orphan HK and RR genes have been found in the *Mycobacterium tuberculosis* (Mtb) genome. SenX3-RegX3 was among the first 2CRS to be identified in Mtb [9,10]. The *senX3-regX3* operon is required for Mtb survival during murine infection [11-13]. However, conflicting results have been reported for the virulence of strains deficient in *senX3* or *regX3* in the lungs and spleens of BALB/c mice [13]. Unlike the case in other 2CRS, expression of *senX3* and *regX3* is monocistronic as well as polycistronic [14,15], raising the possibility that each may function independently of the other under clinically relevant conditions and during host infection. In *M. smegmatis*, co-transcription of *senX3* and *regX3* is required for the expression of phosphate-dependent genes and for optimal growth under inorganic phosphate (P_i_)-limiting conditions [16,17]. Previously, we have shown that the *senX3-regX3* operon is involved in the Mtb phosphate starvation response (PSR) [12], however the individual role of each gene in Mtb survival during P_i_ depletion has not been characterized. Moreover, RegX3, when overexpressed in *M. smegmatis*, can be phosphorylated in the absence of SenX3 in P_i_–rich medium [17], and the SenX3-RegX3 homolog PhoBR in *E. coli* also responds to nutrient starvation in addition to P_i_ depletion [18]. These findings suggest that Mtb SenX3-RegX3 may have a broader role in Mtb virulence beyond the PSR and that these two factors may function independently of each other.

In order to further understand the role of each gene of the *senX3-regX3* 2CRS in Mtb virulence, and in particular the *senX3*-independent contribution of *regX3*, two different mutants deficient in *senX3* or *regX3* were studied under multiple conditions and their phenotypes were compared to the isogenic wild-type parent strain Mtb CDC1551, as well as their respective complemented strains. The expression of *senX3* and *regX3*, as well as that of the phosphate specific transport gene *pstC2*, were evaluated in all the tested strains under physiologically relevant stress conditions. In addition, the growth and survival of each of these strains were assessed during axenic growth in nutrient-rich broth, P_i_ depletion, nutrient starvation, and after aerosol infection in the lungs of BALB/c mice. Our data support the hypothesis that RegX3 may have a SenX3-independent role in Mtb virulence.

## Results

### Confirmation of *senX3::Tn* and *regX3*::Tn complementation

**Figure 1:**
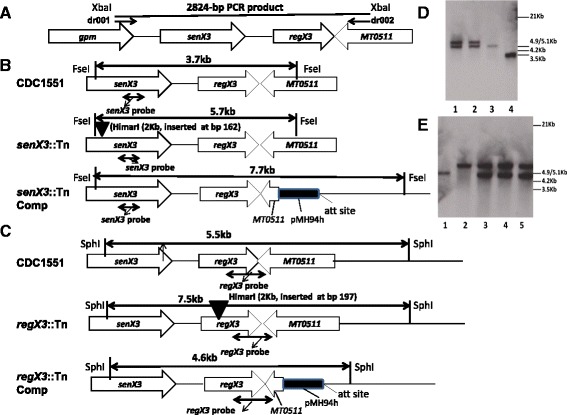
**Genetic confirmation of complementation of** ***senX3*** **::Tn and** ***regX3*** **::Tn. (A)**. Diagram of a 2824-bp fragment containing the *senX3-regX3* operon from the genome of wild-type CDC1551. **(B)** FseI digestion of genomic DNA and hybridization with dig-labeled *senX3* probe reveals fragments measuring 3.7-kb and 5.7-kb in CDC1551 and *senX*3::Tn, respectively, as well as 5.7-kb and 7.7-kb fragments in *senX3*::Tn Comp. **(C)** SphI digestion of genomic DNA followed by hybridization with dig-labeled *regX3* probe reveals 5.5-kb and 7.5-kb fragments in CDC1551 and *regX3*::Tn, respectively, as well as 7.5-kb and 4.6-kb fragments in *regX3*::Tn Comp. **(D)** Southern analysis of *senX3* complementation. Lanes 1 and 2: *SenX3*::Tn Comp candidates with appropriately-sized bands; Lane 3: *SenX*3::Tn; Lane 4: Wild-type CDC1551. **(E)** Southern analysis of *regX3* complementation. Lane 1: Wild-type CDC551; Lane 2: *regX*3::Tn; Lanes 3–5: *regX3*::Tn Comp candidates with appropriately-sized bands.

In order to confirm that phenotypes observed for *senX3*::Tn and *regX3*::Tn were attributable to deficiency of *senX3* and *regX3*, respectively, we complemented each mutant by reintroducing the native gene. Due to the complexity of the operon, which includes monocistronic and bicistronic transcription of each gene [15], each mutant was complemented with the entire *senX3*-*regX3* operon. Successful complementation was confirmed by Southern blot using dig-labeled probes after DNA digestion of complement candidates, as well as the wild-type and mutant strains. As expected, digestion of genomic DNA with FseI and hybridization with dig-labeled *senX3* probe revealed fragments of 5.7-kb and 7.7-kb in the *senX3*::Tn complemented strain (*senX3*::Tn Comp), a 3.7-kb fragment in the wild-type strain, and a 5.7-kb fragment in *senX3*::Tn (Figure 1B and D). SphI digestion of genomic DNA followed by binding of dig-labeled *regX3* probe showed 4.6-kb and 7.5-kb fragments in the *regX3*::Tn complement candidate strains (*regX3*::Tn Comp), a 5.5-kb fragment in the wild-type strain, and a 7.5-kb fragment in *regX3*::Tn (Figure 1C and E). In addition, kanamycin and hygromycin resistance cassettes were PCR-amplified from each of these complemented strains (data not shown). We have shown previously that bicistronic expression of *regX3* was abolished in *senX3*::Tn, although monocistronic expression of the gene is preserved in this strain [15].

### Disruption of *senX3* reduces *regX3* expression during phosphate depletion, but each gene is equally required for regulation of the phosphate-specific transport system

**Figure 2:**
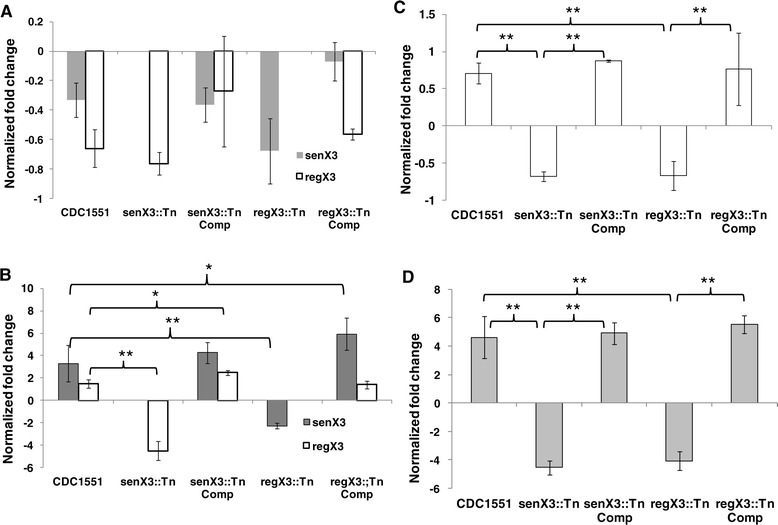
**Gene expression and regulation of** ***senX3*** **,** ***regX3,*** **and the phosphate-specific transport gene** ***pstC2*** **in each strain by qRT-PCR.** An equal amount of total RNA samples were used for cDNA synthesis with random primers. The cycle threshold value (C_T_) of each gene of interest was normalized to that of the housekeeping gene *sigA* to generate ∆C_T_, which was then converted to fold change (1.83e-∆C_T_). **(A)** Expression of the genes *senX3* and *regX3* in Middlebrook 7H9 broth. **(B)** Expression of the genes *senX3* and *regX3* during P_i_ depletion. **(C)** Expression of the *pstC2* gene in Middlebrook 7H9 broth. **(D)** Expression of the *pstC2* gene during P_i_ depletion. The samples were prepared as duplicates and the experiment was repeated twice with similar results. *p <0.05; **p <0.01.

Mildly reduced expression of *senX3* and *regX3* relative to *sigA* was observed in *regX3*::Tn and *senX3*::Tn, respectively, in Middlebrook 7H9 broth compared with the wild type parent strain (Figure 2A; *p* >0.05). Although *regX3* can be expressed independently of *senX3* [15], disruption of the latter gene led to decreased Mtb expression of *regX3* during P_i_ depletion (*p* < 0.01) (Figure 2B). Gene expression levels of *senX3* and *regX3* were restored in *senX3*::Tn Comp and *regX3*::Tn Comp, respectively, during P_i_ depletion, but the cognate genes were overexpressed in each complemented strain relative to the wild type (*p* < 0.05) (Figure 2B), likely due to a second copy of each of these genes in the respective complemented strains. In Middlebrook 7H9 broth, mild down-regulation of *pstC2* expression was observed in *senX3*::Tn and *regX3*::Tn relative to the wild-type strain (*p* < 0.01), and wild-type *pstC2* transcript levels were restored in each of the complemented strains (Figure 2C). Each mutant showed similar, significant down-regulation of the P_i_-specific transport gene *pstC2* relative to the wild-type strain during P_i_ depletion (*p* < 0.01), and wild-type expression levels of the gene were restored in each complemented strain (Figure 2D). Disruption of *senX3* led to decreased expression of *regX3* during nutrient starvation (*p* < 0.05). However, wild-type levels of *senX3* and *regX3* expression were not observed in *senX3*::Tn Comp and *regX3*::Tn Comp, respectively, although *senX3* expression was partially restored in *regX3*::Tn Comp under this condition (data not shown). The abundance of *pstC2* transcripts was not significantly altered in either mutant relative to wild type during nutrient starvation (*p* > 0.05).

### *senX3* and *regX3* are equally required for Mtb growth during P_i_ depletion

**Figure 3:**
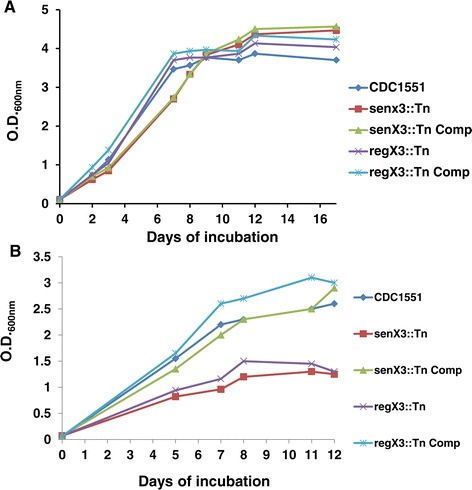
**Mtb** ***senX3*** **and** ***regX3*** **are required for optimal growth during P**_**i**_ **depletion, but not in P**_**i**_ **-replete broth.** The wild-type, *senX3*::Tn, *senX3*::Tn Comp, *regX3*::Tn, and *regX3*::Tn Comp strains were each cultured in reconstituted 7H9 broth containing 25 mM P_i_ **(A)** and P_i_-depleted broth (50 μM P_i_) **(B)**. Each experiment was repeated twice with similar values of optimal density (O.D.600_nm_).

The growth kinetics of each mutant and its respective complement, together with the isogenic wild-type strain, were studied during P_i_ depletion (50 μM P_i_) and in reconstituted, P_i_-replete 7H9 broth (25 mM P_i_). Each strain showed equivalent growth in P_i_-replete 7H9 broth (Figure 3A), suggesting that *senX3* and *regX3* are dispensable for axenic Mtb growth when P_i_ is abundant. However, *senX3*::Tn and *regX3*::Tn showed a marked growth defect and premature entry into stationary phase relative to the isogenic wild-type and their respective complemented strains during P_i_ depletion (Figure 3B). No obvious difference in growth was observed between the mutants.

**Figure 4:**
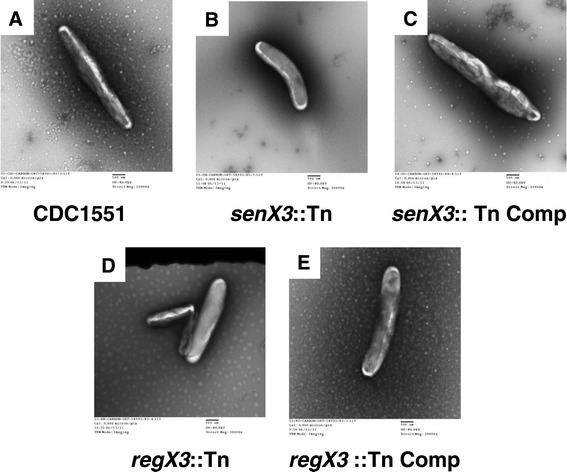
**The contribution of** ***senX3*** **and** ***regX3*** **to bacillary elongation during P**_**i**_ **depletion.** Transmission electron microscopy (TEM) was used to compare the morphology of all the strains after growth in reconstituted 7H9 broth containing 50 μM P_i_ for 21 days. The images are representative of the following strains: **A**. Wild type; **B**. *senX3*::Tn; **C**. *senX3*::Tn Comp; **D**. *regX3*::Tn; **E**. *regX3*::Tn Comp (20,000x magnification).

Previously, we have shown that Mtb bacilli undergo elongation following P_i_ depletion [12]. In order to determine the effect of *senX3* or *regX3* deficiency on Mtb morphology during P_i_ depletion, we used transmission electron microscopy to capture images of at least 40 bacilli of each strain following 21 days of P_i_ depletion or during exponential growth in reconstituted, P_i_-replete 7H9 broth. We found that the mean length of P_i_-depleted *senX3*::Tn and *regX3*::Tn bacilli was 2.62 ± 0.72 and 2.96 ± 0.69, respectively, while the mean length of isogenic wild-type bacilli was 4.02 ± 1.10 (*p >* 0.05). The mean length of the *senX3*::Tn and *regX3*::Tn complement bacilli was 4.02 ± 1.36 and 3.76 ± 0.85, respectively (*p* > 0.05) (Figure 4A-E). The mean length of *senX3*::Tn bacilli (2.48 ± 0.48) and *regX3*::Tn bacilli (2.44 ± 0.72) was equivalent to that of wild-type bacilli (2.46 ± 0.60) during exponential growth in P_i_-replete (25 mM P_i_) broth (*p* > 0.05).

### Higher survival of *senX3*::Tn relative to *regX3*::Tn during nutrient starvation

**Figure 5:**
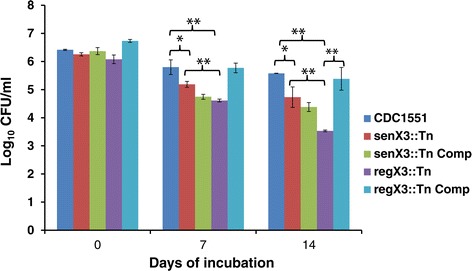
**Both** ***senX3*** **and** ***regX3*** **are required for Mtb survival during nutrient starvation, although there appears to be a** ***senX3*** **-independent contribution of** ***regX3*** **.** The wild-type CDC1551, *senX3*::Tn, *senX3*::Tn Comp, *regX3*::Tn, and *regX3*::Tn Comp were subcultured in 1xPBS with 0.05% Tween-80 and CFU were counted at different time points after plating the diluted cultures on Middlebrook 7H10 plates (mean ± SD) and incubating for 21 days. Triplicate samples were used in the experiment and the experiment was repeated twice under the same condition. *p <0.05; **p <0.01.

Since both monocistronic and bicistronic expression of *regX3* is observed in nutrient-starved Mtb [15], we next studied the requirement for each type of gene expression during nutrient starvation. After 7 days of nutrient starvation, *senX3*::Tn and *regX3*::Tn showed a significant survival defect relative to wild type (*p* < 0.05 and *p* < 0.01, respectively), which was complemented in *regX3*::Tn Comp but not in *senX3*::Tn Comp (Figure 5). At this time point, *senX3*::Tn, in which monocistronic expression of *regX3* is maintained [15], showed higher survival relative to *regX3*::Tn (*p* < 0.01). By 14 days after nutrient starvation, *regX3*::Tn showed markedly reduced survival relative to wild type (*p* < 0.01) and *senX3*::Tn (*p <* 0.01). The survival defect at 14 days was almost completely reversed in *regX3*::Tn Comp (*p <* 0.01 relative to the mutant).

### Higher survival of *senX3*::Tn relative to *regX3*::Tn during chronic infection in mouse lungs

**Figure 6:**
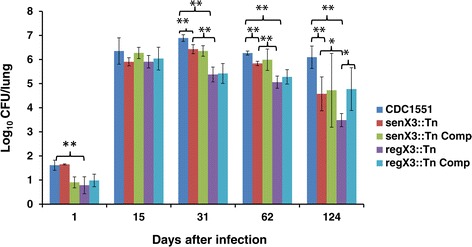
**Both** ***senX3*** **::Tn and** ***regX3*** **::Tn are attenuated, but there appears to be a** ***senX3*** **-independent contribution of** ***regX3*** **for long-term Mtb survival in mouse lungs.** Separate groups of BalB/c mice (4–5 mice per group) were aerosol-infected with the wild-type CDC1551, *senX3*::Tn, *senX3*::Tn Comp, *regX3*::Tn, and *regX3*::Tn Comp. *p <0.05; **p <0.01.

In order to evaluate the potential *senX3*-independent role of *regX3* on Mtb virulence *in vivo*, we aerosol-infected mice with wild type, *senX3*::Tn, *regX3*::Tn, and their respective complemented strains. We observed no significant growth defect for the two mutants during the first 15 days post-infection relative to the wild-type (Figure 6). By Day 31, the wild-type strain achieved a peak bacterial CFU of 6.89 ± 0.13 log_10_ in the lungs, and a reduction in mean lung CFU of 0.5 log_10_ (*p* < 0.01) and 1.51 log_10_ (*p <* 0.01) was observed in *senX3*::Tn and *regX3*::Tn, respectively. By Day 124, mice infected with *senX3*::Tn and *regX3*::Tn had significant reductions in lung bacillary loads of 1.6 log_10_ CFU (*p* < 0.01) and 2.6 log_10_ CFU (*p <* 0.01) relative to mice infected with the wild-type strain. Interestingly, as in the case of nutrient starvation, consistently and significantly higher lung bacillary burdens were observed in mice infected with *senX3*::Tn compared to those in mice infected with *regX3*::Tn at Day 31 (*p* < 0.01), Day 64 (*p* < 0.01), and Day 124 (*p =* 0.05) post-infection (Figure 6). In fact, at Day 124, the lungs of mice infected with *senX3*::Tn had more than 10-fold higher mean CFU relative to those of mice infected with *regX3*::Tn (*p* = 0.05). This lung survival defect was partially restored in *regX3*::Tn Comp (*p* = 0.05 relative to *regX3*::Tn; Figure 6).

### *senX3* and *regX3* contribute to Mtb-induced lung pathology

**Figure 7:**
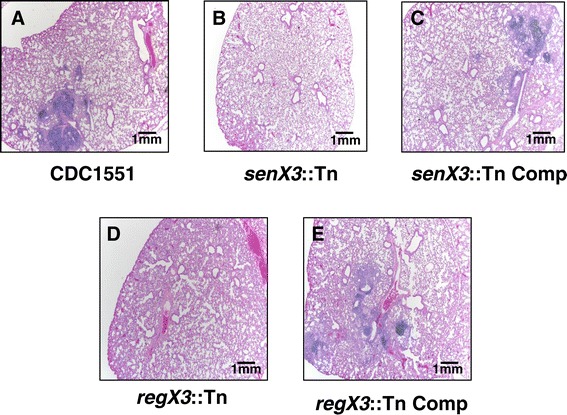
**Histological evaluation of infected mouse lungs at Day 124 post-infection.** Hematoxylin-eosin stains, 200x magnification. **A**. CDC1551; **B**. *senX3*::Tn mutant; **C**. *senX3*::Tn Comp; **D**. *regX3*::Tn; **E**. *regX3*::Tn Comp.

Histopathological examination of mouse lung samples at Day 124 revealed a patchy lymphocytic bronchiolitis with varying extension of the inflammation into the adjoining airways characterized by intra-alveolar macrophages and lymphoid aggregates (Figure 7A-E and Table 1). Bronchiolar obliteration and coalescence of bronchioles was not identified. A perivascular lymphocytic infiltrate was also noted in some of the cases. Lung inflammation was less pronounced in animals infected with *senX3*::Tn and *regX3*::Tn compared with the wild-type strain and their respective complements (Figure 7 A-E). One-way analysis of variance (ANOVA test) across strains showed a statistically significant difference of means in percent inflamed bronchioles (p < 0.05) (Table 1). However, the small number of cases examined precludes a definitive assessment of statistical significance. Acid-fast staining revealed no major differences between groups (data not shown).

**Table 1:** **Histological examination of Mtb-infected mouse lungs**

**Strain**	**Peribronchiolar inflammation**	**Degree of inflammation**	**Extension of inflammation into airway**	**Lymphoid aggregates**	**Bronchioles inflamed**	**No. of bronchioles per slide**	**% of inflamed bronchioles**
CDC1551	yes	mild to mod	no	yes	3.75	37.25	10
*senX3*::Tn	no	none	no	n/a	0.25	45	1
*senX3*::Tn Comp	yes	mild to mod	yes	yes	6.75	40.75	17
*regX3*::Tn	no	none	no	n/a	3.25	36.75	9
*regX3*::Tn Comp	yes	mild	yes	yes	7.6	40.2	19

## Discussion

SenX3-RegX3 is among the best characterized 2CRS in Mtb [9,10], yet many questions remain about its role in virulence. Homologs of *senX3* and *regX3* are found in the genomes of various mycobacterial species, indicating that this evolutionarily conserved 2CRS may play a fundamental regulatory role in mycobacterial physiology [19]. Unlike other 2CRS, in which expression of the operon is bicistronic and auto-regulatory, *senX3* and *regX3* can be transcribed independently of each other [15]. In particular, the intergenic region between the two genes contains several mycobacterium interspersed repetitive units (MIRU), which exist only in *M. leprae* and members of the Mtb complex [9,14,17,20,21], and which can drive *senX3*-independent expression of *regX3* [15]. In the current study, we sought to investigate the *senX3-*independent contribution of *regX3* to Mtb gene regulation, growth, and survival under various physiologically relevant growth-limiting conditions *in vitro* and *in vivo*.

We found that the genes *senX3* and *regX3*, while dispensable for Mtb growth in nutrient-rich broth, are equally required for optimal growth and morphological response during P_i_ depletion. In addition, as evidenced by the statistically greater survival of *senX3*::Tn relative to *regX3*::Tn, we found that *regX3* contributes to Mtb survival independent of *senX3* during prolonged nutrient starvation and chronic infection in mouse lungs.

Although SenX3-RegX3 has been implicated in the mycobacterial PSR [12,16,17], the functional role of each gene in this operon has not been characterized previously during P_i_ depletion. P_i_ depletion is believed to be a physiologically relevant microenvironment encountered by Mtb within the arrested macrophage phagolysosome [22]. Mtb *senX3-regX3* is upregulated in response to P_i_ depletion and expression of the phosphate-specific transport operon *pstS3-pstC2-pstA1* is RegX3-dependent [12]. Previously, we have shown monocistronic upregulation of *senX3* and *regX3* during P_i_ depletion [15], which differs from *M. smegmatis,* in which the *senX3-regX3* operon is co-transcribed. In the current study, we found decreased expression of *regX3* in the *senX3*-deficient mutant relative to the wild type during P_i_ depletion, which could be restored by reintroduction of *senX3*. Decreased *regX3* expression in P_i_-depleted *senX3*::Tn may be due to decreased co-transcription with *senX3* or down-regulation of independently expressed *regX3*. Although the *senX3* promoter is autoregulatory and activated by RegX3, which is phosphorylated by SenX3 [23], it is unknown whether the intergenic region driving independent *regX3* expression is also RegX3-dependent, and, hence, SenX3-dependent.

We found that Mtb mutants deficient in *senX3* or *regX3* exhibited similar growth defects during P_i_ depletion. Despite expression of *regX3* in the *senX3*-deficient mutant, it appears that RegX3 was not functional, as expression of the RegX3-dependent, P_i_-specific transport gene *pstC2* was dramatically downregulated in this strain. Our results are consistent with monocistronic, P_i_-dependent expression of each gene and the putative role of SenX3 as a P_i_ sensor [23]. Therefore, although monocistronic expression of *regX3* is preserved in *senX3*::Tn [15], the cognate HK SenX3 may be required to phosphorylate and activate RegX3 during P_i_ depletion, thereby triggering the PSR. *In vitro* studies in many bacteria have provided evidence in favor of the specificity of 2CRS, in which the HK show a remarkable kinetic preference for their cognate RR primarily at the level of molecular reorganization [24-31]. For example, in *B. subtilis* the HK KinA can phosphorylate either Spo0F or Spo0A, but with more than 50,000-fold preference for Spo0F during sporulation [24,25].

Interestingly, despite similar growth phenotypes of *senX3*::Tn and *regX3*::Tn during P_i_ limitation, the *senX3*-deficient mutant showed markedly higher survival relative to the *regX3*-deficient mutant upon prolonged nutrient starvation and in the lungs of mice, while both mutants were attenuated compared with the wild-type. Our gene expression results demonstrate reduced but detectable expression of *regX3* in the *senX3*-deficient mutant. This discordance in phenotypes suggests the possibility that independently transcribed *regX3* may be translated and phosphorylated in the absence of SenX3, thereby initiating transcription of the RegX3 regulon [15,17,23]. In *M. smegmatis*, the essential gene *regX3* can be activated in the absence of the nonessential gene *senX3* in P_i_-replete broth, suggesting the existence of alternative mechanisms of RegX3 phosphorylation [17]. Similarly, in *E. coli* the RegX3 homologue PhoB can be phosphorylated by the HK EnvZ [32], CreC (PhoM), or acetyl phosphate in the absence of PhoR in P_i_-independent pathways [33]. Precedents for phosphorylation of RR by noncognate HK are also present in Mtb. Thus, the Mtb dormancy response regulator DosR can be phosphorylated by its cognate HK DosS or by the orphan HK DosT [34,35]. Although 2CRS usually maintain specificity to prevent unwanted cross-talk [24-31], significant sequence and structure similarity in HK and RR, as well as the existence of small molecule phosphodonor acetyl-phosphate make cross-regulation possible [36-38]. In addition, cross-regulation could occur at the transcriptional level [39,40]. Under certain circumstances many bacteria appear to employ cross-regulation in order to integrate multiple signals or diversify the response to a single input [4,41]. Therefore, based on our initial findings, we speculate that although Mtb *senX3-regX3* 2CRS maintains specificity during P_i_ depletion, cross-regulation of RegX3 may facilitate a rapid response of Mtb to a changing environment *in vitro* and *in vivo* under other stresses, in which the identity of alternative HK and/or other activating factors remains to be determined. Alternatively, independently transcribed *RegX3* may be functional in the absence of phosphorylation. For example, the *Pseudomonas aeruginosa* RR AlgB and AlgR do not require phosphorylation by the cognate HK KinB for alginate production *in vivo*, indicating that these RR may mediate activation of gene expression through alternative mechanisms [5]. We speculate that the SenX3-RegX3 2CRS may provide a common means by which to transduce the signals of P_i_ depletion and nutrient starvation, leading to induction of the stringent response, which is critical for Mtb persistence in the host [42,43]. Our findings are consistent with the function of the homologous PhoBR 2CRS in *E. coli*, which regulates the transcriptional response to P_i_ depletion and nutrient starvation [18,44]. The mechanism of *regX3* regulation during nutrient starvation and *in vivo* infection remains to be identified.

Higher survival of *senX3*::Tn relative to *regX3*::Tn during chronic infection in mice is consistent with previous data showing higher survival of a *senX3* deletion mutant relative to a *regX3* deletion mutant in mouse spleens [13]. However, our data conflict with those of Rickman et al. in that they showed greater attenuation of the *senX3* deletion strain in mouse lungs relative to the *regX3* deletion strain. These discrepant findings may be due to methodological differences between our study and that by Rickman et al. The latter study used H37Rv, in which the *senX3-regX3* IR comprises three identical 77-bp MIRU rather than two 77-bp MIRU and one 53-bp MIRU, as in the CDC1551 strain used in our study [13,14]. In addition, Rickman et al. used intravenous infection rather than aerosol infection, although both studies used BALB/c mice. Nevertheless, these two studies corroborate our recent finding that *senX3* and *regX3* do not simply function as a bicistronic operon, as previously thought [14], since, in such a case, disruption of *senX3* would be expected to yield mutant phenotypes identical to those of a *regX3*-deficient strain. In the current study, both complemented strains restored wild-type growth and survival during P_i_ depletion, and, in the case of *regX3*::Tn Comp, during nutrient starvation and in the lungs of mice. However, unlike in a previous study [13], our complemented strains failed to restore the wild-type phenotype during mouse infection. There are several potential explanations for these discrepant findings. In comparison to the cloning strategy by Rickman et al., our *senX3-regX3* complementation construct lacked a 211-bp sequence upstream of the *senX3* gene, which may have altered regulation of this gene during the *in vitro* stress conditions studied and mouse infection. Another difference between the two complementation approaches is that in the earlier study the *senX3* and *regX3* genes were fused together by deleting the intergenic region [13]. On the other hand, each of our complemented strains included this intergenic region, since we recently described the potential importance of its promoter activity in driving *senX3-*independent expression of *regX3* [15]. The precise role of this intergenic region in regulating expression of each of these genes requires further study. In addition, the presence of a second copy of the *regX3* gene may have had a deleterious effect on *in vitro and in vivo* survival. As in the case of other defective complemented strains, the chromosomal location at the recombination site may have affected proper expression of *senX3* and *regX3*. Alternatively, the development of other occult mutations during genetic construction of these strains may have contributed to their reduced virulence relative to the wild-type strain. However, deficiency of *senX3* or *regX3* was reported previously to be associated with reduced Mtb virulence in mice, and the attenuated mutant phenotypes were fully complemented in the study by Rickman et al. [13]. Therefore, we favor the explanation that our complementation strategy, as described above, contributed to the incomplete restoration of the wild-type phenotypes in the complemented strains. The mechanisms by which *senX3* and *regX3* are regulated under different stress conditions require further characterization.

## Conclusion

Our data raise the interesting possibility that differential regulation of *senX3* and *regX3* may have evolved to allow for simultaneous coordination of the Mtb PSR [12], while providing a common means by which bacilli may respond to a variety of different physiologically relevant stress conditions. In particular, the mechanisms underlying the SenX3-independent contribution of RegX3 to Mtb virulence deserves further investigation.

## Methods

### Growth conditions and bacterial strains

Supplemented Middlebrook 7H9 broth (7H9 broth, Difco), reconstituted 7H9 broth containing 50 μM P_i_ (P_i_ depletion) [12], and 1xPBS (Biological Quality) containing 0.05% Tween-80 (nutrient starvation) [45] were used to study growth kinetics and bacterial survival. *Mycobacterium tuberculosis* CDC1551 [46] was used as the wild-type strain in all experiments. Mtb strains deficient in *Rv0490/MT0509*/*senX3* (*senX3*::Tn; Tn insertion at bp 162/1233) and *Rv0491*/*MT0510*/*regX3* (*regX3*::Tn; Tn insertion at bp 197/693) were generated previously by mutagenesis of Mtb CDC1551 with the Himar1 transposon (Tn) [47]. The Tn insertion in the *senX3*::Tn mutant is expected to abrogate histidine kinase function, since the phospho-acceptor domain is located between bp 465–657. Similarly, disruption of *regX3* phospho-receiver and DNA-binding domains at bp 21–345 and bp 459–684, respectively, is expected by the Tn insertion in the *regX3*::Tn mutant.

### Complementation of *senX3::Tn* and *regX3::Tn* mutant strains

The *E. coli-*mycobacterium shuttle vector pMH94 [48] was used for complementation of *senX3*::Tn and *regX3*::Tn. A 2,824-bp DNA fragment containing the entire *senX3-regX3* coding region, including 324-bp of 5’ flanking sequence and 378-bp of 3’ flanking sequence was PCR-amplified from Mtb CDC1551 genomic DNA using primers dr001 and dr002 (Table 2 and Figure 1A), which introduced an XbaI site at the 5’ end of each PCR product. After digestion with XbaI, the 2,824-bp PCR product was ligated with similarly digested pMH94, yielding psenX3-regX3, which was transferred into *E. coli* DH5α competent cells, followed by plating on hygromycin-containing LB agar plates. After confirmation by DNA sequencing, the construct was electroporated separately into *senX3::Tn* and *regX3::Tn* competent Mtb cells. Individual transformant colonies were selected on 7H10 agar plates containing kanamycin (25 μg/ml) and hygromycin (50 μg/ml).

**Table 2:** **List of primers used in this study**

**Primer**	**Sequence (5’—3’)**	**Purpose of amplification**
dr001-F	CCCTCTAGAACGAAATCGTCGGACTGAAC	*senX3-regX3* operon and flanking sequence
dr002-R	CCCTCTAGAAGGTGAGATCGCCAAGCTAC	
*senX3*-F	CCGAGTTGATCGAGCTATCC	*senX3* probe and gene expression
*senX3*-R	AGTGCGGTAACCAGCAGAGT	
dl15-F	GCTGACGACTACGTGACCAA	*regX3* probe
dl16-R	CGACGAGCAGCTTTGTCC	
kan-F	CTGTGCTCGACGTTGTCACT	kanamycin gene
kan-R	AGCCAACGCTATGTCCTGAT	
hyg4-F	AGGTCTTCCCGGAACTGCTG	hygromycin gene
hyg4-R	TCCTCGAACACCTCGAAGTC	
*regX3*-F	TGTTGATTGTGGAGGACGAG	gene expression
*regX3*-R	CGCAACTGCTTGCATACATC	
*pstC2*-F	GTCGATCATCTTCGGGTTGT	gene expression
*pstC2*-R	ATTTGGATCAGCGGAGTCTG	

Genomic DNA was purified from *senX3*::Tn and *regX3*::Tn complemented strain candidates. Genetic complementation of *senX3*::Tn and *regX3*::Tn was first confirmed by PCR. Specifically, the kanamycin gene from each transposon insertion was PCR-amplified using the primer pair kan-F/kan-R and the hygromycin gene from the shuttle vector was amplified using hyg4-F/hyg4-R. The sequences of each primer pair are listed in Table 2. In order to further confirm complementation of *senX3*::Tn and *regX3*::Tn, Southern blotting was performed using DIG High Prime DNA Labeling and Detection Starter Kit I according to the manufacturer’s protocol (Roche). Genomic DNA from *senX3*::Tn and *regX3*::Tn complement candidates was digested with FseI and SphI-HF (New England Biolabs), respectively, and electrophoresis was performed on 1% agarose gels. Following denaturation and neutralization, the gels were transferred onto positively charged nylon membranes (GE Healthcare). DNA fragments containing the se*nX3* and *regX3* genes on the membranes were detected by using digoxigenin(dig)-labeled *senX3* and *regX3* probes, respectively, which were generated by adding dig-dUTP in PCR reactions containing primer pairs senX3-F and senX3-R (yielding a 196-bp fragment of the *senX3* gene) or primer pairs dl15 and dl16 (yielding a 576-bp fragment of the *regX3* gene) (Table 2). After pre-hybridization, the membranes were hybridized with dig-labeled *senX3* or *regX3* probes at 68°C overnight, prior to addition of anti-digoxigenin-AP. After stringent washes, the membranes were incubated with the chemiluminescence substrate CSPD (Roche) and exposed on X-ray film at room temperature. Finally, the films were developed in a dark room (AFP imaging).

### Gene expression by quantitative RT-PCR (qRT-PCR)

Total RNA from each Mtb strain was purified from nutrient- and P_i_-starved cultures after 24 hr followed by DNase treatment to remove contaminating DNA [49,50]. Gene expression levels were detected using the primer pairs listed in Table 2 and iCycler 5.0 (Bio-Rad). cDNA was synthesized with random hexamers (Invitrogen) using an equal amount of total RNA samples, which was subjected to two technical replicates of PCR amplification and then averaged to generate a single value for each biological replicate. The cycle threshold value (C_T_) measured for each gene was normalized to that of the house-keeping gene *sigA* under each condition and then converted to fold change (1.83e-∆C_T_) [15,51]. *senX3* expression was determined by primer pair *senX3*-F/*senX3*-R, which amplified from bp 635 to bp 830 of the *senX3* gene, distal to the transposon insertion site (bp 162). *regX3* expression was studied using primer pair *regX3*-F/*regX3*-R, which amplified from bp 20 to bp 212, across the transposon insertion site of the *regX3* gene (bp 197). The samples were prepared as duplicates and statistical analysis was performed using two biological replicates for each sample.

### Growth kinetics and survival during in vitro growth conditions

For kinetics studies during inorganic P_i_ depletion, the pellet of each strain was washed with reconstituted Middlebrook 7H9 broth containing 50 μM P_i_, resuspended in 10 ml (final O.D._600nm_ ~0.05) of the same broth in conical tubes, and incubated at 37°C in a roller incubator. O.D._600nm_ values for each strain were measured by portable spectrometer (Biorad) at each time point up to 12 days. The growth kinetic of each strain in Middlebrook 7H9 broth (Difco) was observed up to 17 days. The experiment was repeated twice under the same growth conditions.

For nutrient starvation conditions, the bacterial pellet of each strain was washed with 1xPBS (0.05%Tween-80) three times followed by re-suspension in 3 ml (final O.D._600nm_ ~0.1) of 1xPBS (0.05%Tween-80) in a 15-ml conical tube prior to standing incubation at 37°C. Colony-forming unit (CFU) counts were assessed at Day 1, Day 7, and Day 14 by plating the culture on Middlebrook 7H10 agar after serial dilutions (Fisher). All the samples were prepared as triplicates and the experiment was repeated twice.

### Transmission electron microscopy (TEM)

Fixed phosphotungstate (PTA) negative stain for electron microscopy study was used to evaluate bacterial morphology [12]. Bacterial cultures were fixed and viewed (Hitachi H-7600 TEM [80 kV]), followed by digital capture (direct magnification, ×20,000). The length of each strain was measured using ImageJ software.

### Animal infections

All animal procedures followed protocols approved by the JHU Animal Care and Use Committee. Female BALB/c mice (4–6 weeks; Charles River) were housed in a Biosafety Level-3, specific pathogen-free facility and fed water and chow *ad libitum*. Separate groups of mice were aerosol-infected using the Inhalation Exposure System (Glas-Col, Terre Haute, IN) calibrated to deliver ~10^2^ bacilli of one of the following Mtb strains: CDC1551, *senX3*::Tn, *regX3*::Tn, *senX3*::Tn complement, or *regX3*::Tn complement. Groups of 4 mice in each group were sacrificed on Days 1, 15, 31, 62, and 124 post-infection. Lungs were removed aseptically and homogenized, and organ homogenates were diluted and plated on supplemented Middlebrook 7H11 plates to determine CFU counts (Fisher).

The upper lobe of the left lung was processed for histological evaluation [52]. Lung sections were examined for the presence or absence of bronchiolitis, bronchiolar obliteration, granulomatous inflammation, and coalescence or confluence of inflamed bronchioles. The type and predominance of inflammatory cell type (lymphocyte, macrophage, or neutrophil) was noted. For each slide both the total number of bronchioles and the number of inflamed bronchioles were counted. Sections were also examined for the presence or absence of perivascular inflammation.

### Statistical analysis

Means and standard deviations were calculated for each data set. Differences between calculated means were compared by the Student’s *t* test in all experiments except for histology studies, where the difference of means in percent inflamed bronchioles was compared by ANOVA test. A p-value ≤ 0.05 was considered statistically significant.

## Abbreviations

Mtb: *Mycobacterium tuberculosis*
2CRS: Two component regulatory system
HK: Histidine kinase
RR: Response regulator
P_i_: Phosphate
PSR: Phosphate starvation response
ANOVA: One-way analysis of variance
MIRU: Mycobacterial interspersed repetitive unit
C_T_: Cycle threshold value
CFU: Colony-forming unit
PTA: Phosphotungstate
TEM: Transmission electron microscopy
Dig: Digoxigenin
qRT-PCR: Quantitative reverse transcriptase PCR
